# Supplementary material for: Development and Psychometric Testing of EPAT‐16: A Short and Valid Measure for Patient‐Centeredness From the Patient's Perspective
Source: Health Expect. 2025 May 20;28(3):e70296. doi: 10.1111/hex.70296 (PMC12090203; doi:10.1111/hex.70296)
Supplement: Supplementary file 2 — Appendix 2 Sample flow chart and characteristics. [file HEX-28-e70296-s003.pdf]

Appendix 2: Sample flow chart and characteristics

Article: Development and psychometric testing of EPAT-16: A short and valid measure for patient-centeredness from the patient's perspective

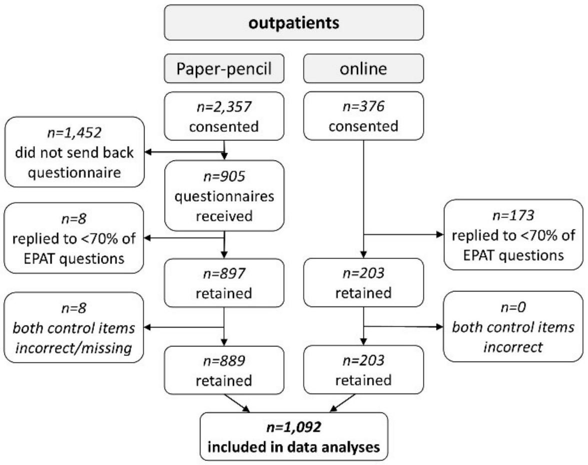

Figure 1: Sample flow chart (Originally published in Christalle et al., 2024[1], licensed under Creative Commons Attribution Non Commercial (CC BY-NC 4.0))

Table 1: Sample characteristics (Originally published in Christalle et al., 2024[1], licensed under Creative Commons Attribution Non Commercial (CC BY-NC 4.0))

Characteristics	Outpatients	Inpatients
Total sample size	n = 1092	n = 931
In treatment for		
Cardiovascular disease	n = 277 (25.4%)	n = 286 (30.7 %)
Cancer	n = 273 (25.0%)	n = 351 (37.7 %)
Musculoskeletal disease	n = 217 (19.9 %)	n = 92 (9.9 %)
Mental disorder	n = 273 (25.0 %)	n = 202 (21.7 %)
No information	n = 52 (4.8%)[+]	n = 0 (0 %)
Age (in years)	M = 53.1 (SD = 17.5)	M = 56.0 (SD = 17.6)
No response	n = 23 (2.1%)	n = 19 (2.0 %)
Years since initial diagnosis	M = 11.4 (SD = 12.0)	M = 8.5 (SD = 10.0)
No response	n = 124 (11.4%)	n = 118 (12.7 %)
Years as patient in this outpatient clinic	M = 4.53 (SD = 6.5)	-
No response	n = 129 (11.8%)	-
Length of stay (in days)	-	M = 18.1 (SD = 30.6)
No response	-	n = 74 (7.9 %)
Health literacy[a]	M = 50.7 (SD = 7.7)	M = 50.6 (SD = 7.7)
No response	n = 439 (40.2%)	n = 304 (32.7 %)
Satisfaction[b]	M = 27.5 (SD = 4.6)	M = 28.0 (SD = 4.9)
No response	n = 15 (1.4%)	n = 13 (1.4 %)

Characteristics	Outpatients	Inpatients
Health status[c]	M = 3.4 (SD = 2.8)	M = 3.4 (SD = 4.1)
No response	n = 44 (4.0%)	n = 36 (3.9 %)
Comorbidity (Do you have any further diseases?)		
Yes	n = 551 (50.5 %)	n = 467 (50.2 %)
No	n = 450 (41.2 %)	n = 393 (42.2 %)
No response	n = 91 (8.3%)	n = 71 (7.6 %)
Gender		
Female	n = 646 (59.2 %)	n = 384 (41.2 %)
Male	n = 420 (38.5 %)	n = 517 (55.5 %)
Diverse	n = 5 (0.5 %)	n = 6 (0.6 %)
No response	n = 21 (1.9%)	n = 24 (2.6 %)
Marital status		
Unmarried and unpartnered	n = 343 (31.4 %)	n = 237 (25.5 %)
Married or partnered	n = 552 (50.5 %)	n = 524 (56.3 %)
Divorced	n = 107 (9.8 %)	n = 80 (8.6 %)
Widowed	n = 61 (5.6 %)	n = 54 (5.8 %)
No response	n = 29 (2.7%)	n = 36 (3.9 %)
Formal education		
Low[d]	n = 10 (0.9 %)	n = 20 (2.1 %)
Intermediate[e]	n = 404 (37.0 %)	n = 383 (41.2 %)
High[f]	n = 267 (24.5 %)	n = 204 (21.9 %)
Very high[g]	n = 376 (34.4 %)	n = 279 (30.0 %)
No response	n = 23 (2.1%)	n = 27 (2.9 %)
Occupational status*		
Employed	n = 447 (40.9 %)	n = 357 (38.3 %)
Unemployed	n = 66 (6.0 %)	n = 64 (6-9 %)
Student/trainee	n = 91 (8.3 %)	n = 50 (5.4 %)
Parental leave/stay at home	n = 57 (5.2 %)	n = 35 (3.8 %)
Retired	n = 394 (36.1 %)	n = 392 (42.1 %)
No response	n = 26 (2.4%)	n = 17 (1.8 %)
Health insurance*		
Statutory	n = 910 (83.3 %)	n = 721 (77.4 %)
Private	n = 164 (15.0 %)	n = 214 (23.0 %)
No response	n = 32 (2.9%)	n = 34 (3.7 %)
Migration background (Were you or your parents born in another country than Germany?)		
Yes	n = 167 (15.3 %)	n = 150 (16.1 %)
No	N = 898 (82.2 %)	n = 758 (81.4 %)
No response	n = 27 (2.5%)	n = 23 (2.5 %)

Note: * multiple answers possible, M = Mean, SD = Standard Deviation, [+] These patients were recruited in a general practice. They assured us, that they were treated for one of the four health conditions, but failed to indicate which one in the questionnaire. [a] Health literacy measured by the total sum score of the HLS-EU-Q16[2], range 16-64, high value = high health literacy, [b] Satisfaction with care measured by ZUF-8[3], range 8-32, high value = high satisfaction, [b] General health status measured by first item of SF-12[4], range 0-5, high value = low health status, [d] low = no formal degree or graduation after less than 10years at school; [e] intermediate = graduation after 9 or 10years at school; [f] high = graduation after more than 10years at school; [g] very high = college or university degree

References

1. Christalle E, Zeh S, Führes H, et al. Through the patients' eyes: psychometric evaluation of the 64-item version of the Experienced Patient-Centeredness Questionnaire (EPAT-64). *BMJ Quality & Safety* 2024:bmjqs-2024-017434. doi: 10.1136/bmjqs-2024-017434
2. Röthlin F, Pelikan JM, Ganahl K. Die Gesundheitskompetenz der 15-jährigen Jugendlichen in Österreich. *Abschlussbericht der österreichischen Gesundheitskompetenz Jugendstudie im Auftrag des Hauptverbands der österreichischen Sozialversicherungsträger (HVSV)* 2013
3. Kriz D, Nübling R, Steffanowski A, et al. Patientenzufriedenheit in der stationären Rehabilitation: Psychometrische Reanalyse des ZUF-8 auf der Basis multizentrischer Stichproben verschiedener Indikation. *Zeitschrift für medizinische Psychologie* 2008;17(2-3):67-79.
4. Wirtz MA, Morfeld M, Glaesmer H, et al. Konfirmatorische Prüfung der Skalenstruktur des SF-12 Version 2.0 in einer deutschen bevölkerungs-repräsentativen Stichprobe. *Diagnostica* 2018;64(2):84-96.
