# Supplementary material for: Development and Psychometric Testing of EPAT‐16: A Short and Valid Measure for Patient‐Centeredness From the Patient's Perspective
Source: Health Expect. 2025 May 20;28(3):e70296. doi: 10.1111/hex.70296 (PMC12090203; doi:10.1111/hex.70296)
Supplement: Supplementary file 3 — Appendix 3 Item characteristics per medical condition. [file HEX-28-e70296-s005.pdf]

Appendix 3: Item characteristics per medical condition

Article: Development and psychometric testing of EPAT-16: A short and valid measure for patient-centeredness from the patient's perspective

Abbreviations:

- Card = Cardiovascular diseases
- Mus = Muscoloskeletal diseases
- Ment = Mental disorders

Table 1: Mean and standard deviation per medical condition – outpatient sample

		mean					standard deviation				
	Item	**All**	Card	Cancer	Mus	Ment	**All**	Card	Cancer	Mus	Ment
Item 1	The healthcare professionals were sensitive (for example they addressed my feelings, showed understanding, or empathized with my situation).	**5.1**	5.1	5.1	4.8	5.1	**1.2**	1.2	1.2	1.5	1.2
Item 2	I trusted my healthcare professionals.	**5.2**	5.3	5.3	5.0	5.3	**1.1**	1.1	1.0	1.3	1.0
Item 3	My wishes, needs and expectations were asked and taken into account in the treatment.	**4.8**	4.7	4.7	4.5	4.7	**1.4**	1.4	1.4	1.6	1.4
Item 4	My entire personal life was taken into account during the treatment (for example, job, family and friends, partnership and sexuality, culture and religion, age, or financial circumstances).	**3.9**	3.2	3.5	3.4	3.5	**1.8**	1.8	1.8	1.8	1.8
Item 5	I was given enough time to describe my concerns and my situation (for example, medical history or current symptoms).	**5.3**	5.3	5.4	5.1	5.4	**1.1**	1.2	0.9	1.3	0.9
Item 6	I was asked if I use or would like to use additional services (for example, support groups, counseling, health courses, complementary and alternative medicine, or spiritual support/pastoral care).	**3.1**	2.3	3.0	2.6	3.0	**2.0**	1.9	1.9	1.8	1.9
Item 7	The processes within the team were well organized.	**5.1**	5.0	5.2	5.2	5.2	**1.0**	1.1	1.0	0.9	1.0
Item 8	If I wanted to speak to a physician, they were easily accessible.	**4.7**	4.6	5.0	4.3	5.0	**1.4**	1.4	1.0	1.6	1.0
Item 9	It was discussed with me whether follow-up appointments would be useful (for example, for aftercare or further treatment).	**5.1**	5.2	5.2	4.8	5.2	**1.4**	1.3	1.2	1.6	1.2
Item 10	I was encouraged to speak up if I noticed inconsistencies in my treatment.	**3.9**	3.7	4.1	3.5	4.1	**1.8**	1.9	1.8	1.9	1.8
Item 11	I received information about my condition from my healthcare professionals (for example, causes, symptoms, effects or course).	**4.6**	4.6	4.8	4.3	4.8	**1.6**	1.6	1.4	1.6	1.4
Item 12	I was an equal partner with my healthcare professionals (for example, in making decisions or sharing information).	**5.0**	5.0	5.0	4.8	5.0	**1.2**	1.1	1.1	1.5	1.1
Item 13	I was informed about the options for involving my family members in the treatment (for example, accompanying to appointments, participating in conversations, or assisting with medication intake).	**3.1**	3.0	3.4	2.2	3.4	**2.0**	2.0	2.0	1.7	2.0
Item 14	I was encouraged to improve my health by changing my behavior (for example, through diet, exercise, reducing tobacco or alcohol).	**4.0**	3.5	3.9	3.9	3.9	**1.8**	1.9	1.8	1.7	1.8
Item 15	When I had pain, I was helped quickly.	**4.8**	5.0	5.3	4.5	5.3	**1.4**	1.4	0.9	1.4	0.9
Item 16	The healthcare professionals addressed my fears and concerns (for example, by showing understanding and providing encouragement).	**4.4**	3.8	4.2	3.9	4.2	**1.7**	1.9	1.6	1.7	1.6
	EPAT-16 sum score	**72.6**	70.9	73.5	68.3	76.6	**16.0**	15.7	14.9	18.2	13.9

Table 2: Further item characteristics per medical condition – outpatient sample

	**Item difficulty**					**'does not concern me'**					**no reply**					**item total correlation**				
	**All**	Card	Cancer	Mus	Ment	**All**	Card	Cancer	Mus	Ment	**All**	Card	Cancer	Mus	Ment	**All**	Card	Cancer	Mus	Ment
Item 1	**82.1**	81.5	82.2	75.2	82.2	**4.2%**	8.3%	4.4%	2.8%	4.4%	**1.1%**	1.4%	1.8%	0.5%	1.8%	**.714**	.652	.672	.805	.680
Item 2	**84.8**	86.0	86.1	79.2	86.1	**1.8%**	4.7%	0.4%	0.9%	0.4%	**1.2%**	1.1%	1.5%	0.9%	1.5%	**.694**	.623	.666	.778	.680
Item 3	**75.1**	74.5	73.3	69.5	73.3	**8.3%**	14.1%	10.6%	3.7%	10.6%	**0.8%**	1.1%	0.7%	1.4%	0.7%	**.748**	.689	.687	.827	.760
Item 4	**57.0**	43.9	49.4	48.6	49.4	**16.1%**	28.9%	18.3%	12.4%	18.3%	**1.2%**	1.8%	1.8%	0.5%	1.8%	**.646**	.609	.680	.689	.637
Item 5	**86.3**	85.9	87.9	81.1	87.9	**2.6%**	5.1%	2.2%	2.3%	2.2%	**1.0%**	2.5%	1.1%	0.5%	1.1%	**.673**	.850	.583	.787	.625
Item 6	**42.3**	26.9	39.5	31.5	39.5	**30.4%**	54.5%	23.8%	29.0%	23.8%	**1.1%**	1.8%	1.8%	0.5%	1.8%	**.493**	.490	.548	.532	.314
Item 7	**82.8**	80.8	83.9	83.6	83.9	**7.1%**	3.6%	3.7%	2.8%	3.7%	**0.8%**	1.8%	0.7%	0.5%	0.7%	**.403**	.415	.369	.429	.418
Item 8	**73.1**	72.6	80.4	66.0	80.4	**23.4%**	30.7%	12.8%	33.6%	12.8%	**1.3%**	1.4%	2.2%	0.5%	2.2%	**.496**	.500	.539	.524	.446
Item 9	**81.7**	83.5	84.6	76.4	84.6	**13.1%**	16.2%	12.8%	11.1%	12.8%	**1.4%**	0.7%	3.3%	0.0%	3.3%	**.479**	.478	.488	.486	.465
Item 10	**57.5**	54.1	61.6	50.6	61.6	**23.4%**	31.0%	18.3%	18.4%	18.3%	**0.8%**	0.7%	1.5%	0.9%	1.5%	**.614**	.523	.645	.695	.569
Item 11	**71.1**	71.1	75.6	66.3	75.6	**10.9%**	15.5%	10.3%	6.5%	10.3%	**0.7%**	1.4%	0.4%	0.9%	0.4%	**.630**	.643	.555	.729	.645
Item 12	**79.4**	80.2	79.5	76.1	79.5	**7.6%**	13.7%	5.1%	6.0%	5.1%	**1.3%**	1.4%	1.1%	1.8%	1.1%	**.665**	.658	.590	.801	.612
Item 13	**41.5**	40.4	49.0	24.4	49.0	**42.6%**	55.2%	30.8%	49.8%	30.8%	**1.3%**	1.8%	2.2%	0.5%	2.2%	**.553**	.536	.583	.581	.502
Item 14	**60.9**	50.7	58.6	58.9	58.6	**26.0%**	40.1%	27.1%	18.9%	27.1%	**0.8%**	1.1%	1.1%	0.9%	1.1%	**.592**	.611	.639	.607	.556
Item 15	**75.8**	79.5	86.0	69.1	86.0	**54.8%**	69.7%	52.7%	19.8%	52.7%	**1.0%**	1.1%	1.8%	0.9%	1.8%	**.551**	.476	.529	.668	.572
Item 16	**67.8**	56.9	63.2	58.6	63.2	**19.2%**	36.1%	18.7%	19.4%	18.7%	**0.8%**	0.7%	1.5%	0.5%	1.5%	**.731**	.688	.721	.788	.744

Notes: item total correlation = Corrected item total correlation with the other EPAT-16 items

Table 3: Mean and standard deviation per medical condition – inpatient sample

		mean					standard deviation				
	Item	**All**	Card	Cancer	Mus	Ment	**All**	Card	Cancer	Mus	Ment
Item 1	The healthcare professionals were sensitive (for example they addressed my feelings, showed understanding, or empathized with my situation).	**5.1**	5.1	5.5	4.6	5.5	**1.2**	1.2	0.8	1.3	0.8
Item 2	I trusted my healthcare professionals.	**5.3**	5.4	5.6	4.9	5.6	**1.1**	1.0	0.8	1.3	0.8
Item 3	My wishes, needs and expectations were asked and taken into account in the treatment.	**4.8**	4.8	5.1	4.3	5.1	**1.3**	1.4	1.2	1.6	1.2
Item 4	My entire personal life was taken into account during the treatment (for example, job, family and friends, partnership and sexuality, culture and religion, age, or financial circumstances).	**3.8**	3.3	3.8	3.5	3.8	**1.8**	1.7	1.8	1.9	1.8
Item 5	I was given enough time to describe my concerns and my situation (for example, medical history or current symptoms).	**5.2**	5.3	5.5	4.8	5.5	**1.1**	1.0	0.9	1.3	0.9
Item 6	I was asked if I use or would like to use additional services (for example, support groups, counseling, health courses, complementary and alternative medicine, or spiritual support/pastoral care).	**3.4**	2.5	3.8	2.8	3.8	**2.0**	1.8	2.0	1.8	2.0
Item 7	The processes within the team were well organized.	**5.1**	5.2	5.4	4.9	5.4	**1.1**	1.1	0.9	1.2	0.9
Item 8	If I wanted to speak to a physician, they were easily accessible.	**4.8**	4.9	5.2	4.5	5.2	**1.2**	1.2	1.0	1.4	1.0
Item 9	It was discussed with me whether follow-up appointments would be useful (for example, for aftercare or further treatment).	**5.0**	5.0	5.3	4.8	5.3	**1.4**	1.4	1.2	1.5	1.2
Item 10	I was encouraged to speak up if I noticed inconsistencies in my treatment.	**4.1**	4.1	4.3	3.7	4.3	**1.7**	1.7	1.8	1.8	1.8
Item 11	I received information about my condition from my healthcare professionals (for example, causes, symptoms, effects or course).	**4.6**	4.8	4.7	4.4	4.7	**1.5**	1.4	1.4	1.7	1.4
Item 12	I was an equal partner with my healthcare professionals (for example, in making decisions or sharing information).	**4.8**	4.8	5.2	4.4	5.2	**1.3**	1.3	1.0	1.5	1.0
Item 13	I was informed about the options for involving my family members in the treatment (for example, accompanying to appointments, participating in conversations, or assisting with medication intake).	**3.3**	3.3	3.4	2.7	3.4	**1.9**	2.0	1.9	1.8	1.9
Item 14	I was encouraged to improve my health by changing my behavior (for example, through diet, exercise, reducing tobacco or alcohol).	**4.0**	3.8	3.8	3.6	3.8	**1.7**	1.8	1.8	1.7	1.8
Item 15	When I had pain, I was helped quickly.	**5.4**	5.6	5.7	4.9	5.7	**1.0**	0.8	0.7	1.3	0.7
Item 16	The healthcare professionals addressed my fears and concerns (for example, by showing understanding and providing encouragement).	**4.4**	4.1	4.7	3.6	4.7	**1.6**	1.7	1.4	1.8	1.4
	EPAT-16 sum score	**73.6**	72.8	77.5	66.9	71.0	**15.3**	14.3	13.5	18.4	16.6

Table 4: Further item characteristics per medical condition - inpatient sample

	**Item difficulty**					**'does not concern me'**					**no reply**					**item total correlation**				
	**All**	Card	Cancer	Mus	Ment	**All**	Card	Cancer	Mus	Ment	**All**	Card	Cancer	Mus	Ment	**All**	Card	Cancer	Mus	Ment
Item 1	**81.9**	82.5	89.0	71.6	89.0	**3.9%**	7.7%	2.3%	5.4%	2.3%	**0.8%**	0.7%	0.9%	1.1%	0.9%	**.701**	.585	.649	.791	.797
Item 2	**86.3**	88.6	91.2	78.7	91.2	**1.2%**	2.4%	0.3%	2.2%	0.3%	**0.4%**	1.0%	0.3%	0.0%	0.3%	**.676**	.537	.639	.804	.758
Item 3	**75.8**	76.9	81.5	65.2	81.5	**5.5%**	9.4%	5.9%	3.3%	5.9%	**0.9%**	1.0%	0.6%	0.0%	0.6%	**.742**	.646	.695	.845	.817
Item 4	**55.8**	45.8	55.3	49.1	55.3	**14.2%**	24.1%	15.8%	4.3%	15.8%	**0.8%**	0.3%	0.9%	2.2%	0.9%	**.593**	.615	.660	.620	.715
Item 5	**85.0**	87.0	89.9	76.5	89.9	**2.8%**	2.1%	5.3%	0.0%	5.3%	**0.5%**	0.3%	0.9%	0.0%	0.9%	**.690**	.616	.645	.750	.750
Item 6	**48.7**	30.0	56.8	35.5	56.8	**25.8%**	43.7%	21.4%	21.7%	21.4%	**1.5%**	1.0%	2.3%	1.1%	2.3%	**.531**	.552	.550	.597	.538
Item 7	**81.8**	83.9	88.6	78.0	88.6	**1.2%**	1.4%	0.3%	3.3%	0.3%	**1.0%**	1.4%	0.6%	1.1%	0.6%	**.605**	.557	.625	.600	.645
Item 8	**76.2**	78.0	83.5	69.5	83.5	**6.6%**	9.8%	5.6%	6.5%	5.6%	**1.0%**	1.0%	1.5%	0.0%	1.5%	**.611**	.539	.605	.708	.607
Item 9	**80.0**	79.9	86.3	75.1	86.3	**7.2%**	8.4%	7.3%	2.2%	7.3%	**1.0%**	1.4%	1.2%	1.1%	1.2%	**.547**	.470	.463	.619	.650
Item 10	**61.9**	62.0	65.5	53.5	65.5	**14.2%**	15.0%	19.1%	8.7%	19.1%	**1.2%**	1.0%	1.5%	1.1%	1.5%	**.610**	.592	.551	.745	.632
Item 11	**72.1**	76.6	73.7	68.1	73.7	**6.4%**	5.2%	7.9%	5.4%	7.9%	**1.3%**	1.4%	1.8%	1.1%	1.8%	**.606**	.578	.606	.719	.627
Item 12	**75.8**	75.1	83.6	68.5	83.6	**4.4%**	7.3%	3.8%	2.2%	3.8%	**1.8%**	2.1%	2.6%	1.1%	2.6%	**.643**	.581	.560	.695	.715
Item 13	**46.0**	45.4	48.7	33.0	48.7	**29.6%**	39.2%	30.5%	28.3%	30.5%	**1.0%**	1.0%	1.2%	0.0%	1.2%	**.578**	.590	.618	.662	.471
Item 14	**60.8**	56.0	56.5	52.6	56.5	**25.8%**	29.0%	34.0%	20.7%	34.0%	**0.5%**	0.3%	1.2%	0.0%	1.2%	**.492**	.484	.577	.565	.537
Item 15	**87.1**	91.3	94.0	77.0	94.0	**19.4%**	23.8%	17.0%	5.4%	17.0%	**0.8%**	1.4%	0.6%	0.0%	0.6%	**.492**	.455	.440	.561	.505
Item 16	**68.8**	62.3	74.5	51.3	74.5	**15.9%**	25.2%	17.9%	14.1%	17.9%	**1.5%**	0.7%	2.6%	1.1%	2.6%	**.688**	.633	.660	.755	.815

Notes: item total correlation = Corrected item total correlation with the other EPAT-16 items
