# Supplementary material for: Development and Psychometric Testing of EPAT‐16: A Short and Valid Measure for Patient‐Centeredness From the Patient's Perspective
Source: Health Expect. 2025 May 20;28(3):e70296. doi: 10.1111/hex.70296 (PMC12090203; doi:10.1111/hex.70296)
Supplement: Supplementary file 4 — Appendix 4 Residual correlations, modification indices and modified model revised. [file HEX-28-e70296-s004.pdf]

Appendix 4: Residual correlations, modification indices and modified model

Article: Development and psychometric testing of EPAT-16: A short and valid measure for patient-centeredness from the patient's perspective

Item abbreviations used in tables below:

Abbreviation	Dimension	Item
Essential char	Essential characteristics of the clinician	The healthcare professionals were sensitive (for example they addressed my feelings, showed understanding, or empathized with my situation).
Relationship	Clinician-patient relationship	I trusted my healthcare professionals.
Unique person	Patient as a unique person	My wishes, needs and expectations were asked and taken into account in the treatment.
Biopsychosocial	Biopsychosocial perspective	My entire personal life was taken into account during the treatment (for example, job, family and friends, partnership and sexuality, culture and religion, age, or financial circumstances).
Communication	Clinician-patient communication	I was given enough time to describe my concerns and my situation (for example, medical history or current symptoms).
Integration	Integration of medical and non-medical care	I was asked if I use or would like to use additional services (for example, support groups, counseling, health courses, complementary and alternative medicine, or spiritual support/pastoral care).
Teamwork	Teamwork and teambuilding	The processes within the team were well organized.
Access	Access to care	If I wanted to speak to a physician, they were easily accessible.
Coordination	Coordination and continuity of care	It was discussed with me whether follow-up appointments would be useful (for example, for aftercare or further treatment).
Safety	Patient safety	I was encouraged to speak up if I noticed inconsistencies in my treatment.
Information	Patient information	I received information about my condition from my healthcare professionals (for example, causes, symptoms, effects or course).
Involvement	Patient involvement in care	I was an equal partner with my healthcare professionals (for example, in making decisions or sharing information).
Family	Involvement of family and friends	I was informed about the options for involving my family members in the treatment (for example, accompanying to appointments, participating in conversations, or assisting with medication intake).
Empowerment	Patient empowerment	I was encouraged to improve my health by changing my behavior (for example, through diet, exercise, reducing tobacco or alcohol).
Physical	Physical support	When I had pain, I was helped quickly.
Emotional	Emotional support	The healthcare professionals addressed my fears and concerns (for example, by showing understanding and providing encouragement).

Table 1: Residual correlations in the unidimensional model – outpatient sample

	Essential char	Relation-ship	Unique person	Biopsycho-social	Communi-cation	Integration	Teamwork	Access	Coordi-nation	Safety	Information	Involvement	Family	Empower-ment	Physical	Emotional
Essential char	0	0.059	-0.005	-0.043	0.021	-0.063	-0.022	-0.001	-0.047	-0.042	-0.028	0.01	-0.092	-0.084	0.01	0.04
Relationship	0.059	0	-0.001	-0.075	0.034	-0.068	-0.011	-0.017	0.01	-0.061	-0.025	0.044	-0.051	-0.095	0.088	-0.034
Unique person	-0.005	-0.001	0	-0.011	0.021	0.013	-0.052	-0.04	-0.021	0.031	0.013	0.037	-0.023	-0.026	-0.041	-0.009
Biopsychosocial	-0.043	-0.075	-0.011	0	-0.077	0.219	-0.024	-0.031	-0.007	0.122	0.013	-0.031	0.194	0.157	-0.049	0.141
Communication	0.021	0.034	0.021	-0.077	0	-0.057	0.03	0.01	0.007	-0.03	-0.009	0.046	-0.095	-0.051	0.022	-0.057
Integration	-0.063	-0.068	0.013	0.219	-0.057	0	0	-0.001	0.01	0.133	0.009	-0.065	0.249	0.213	0.003	0.081
Teamwork	-0.022	-0.011	-0.052	-0.024	0.03	0	0	0.161	0.092	0.038	0.032	-0.017	0.031	0.055	0.048	-0.045
Access	-0.001	-0.017	-0.04	-0.031	0.01	-0.001	0.161	0	-0.022	0.001	0.009	0.033	0.066	0.007	0.058	-0.038
Coordination	-0.047	0.01	-0.021	-0.007	0.007	0.01	0.092	-0.022	0	0.018	0.126	-0.008	0.023	0.035	-0.019	-0.017
Safety	-0.042	-0.061	0.031	0.122	-0.03	0.133	0.038	0.001	0.018	0	0.044	-0.049	0.184	0.117	0.007	0.009
Information	-0.028	-0.025	0.013	0.013	-0.009	0.009	0.032	0.009	0.126	0.044	0	-0.051	0.125	0.123	-0.042	-0.005
Involvement	0.01	0.044	0.037	-0.031	0.046	-0.065	-0.017	0.033	-0.008	-0.049	-0.051	0	-0.115	-0.059	0	-0.037
Family	-0.092	-0.051	-0.023	0.194	-0.095	0.249	0.031	0.066	0.023	0.184	0.125	-0.115	0	0.214	0.062	0.067
Empowerment	-0.084	-0.095	-0.026	0.157	-0.051	0.213	0.055	0.007	0.035	0.117	0.123	-0.059	0.214	0	-0.055	0.074
Physical	0.01	0.088	-0.041	-0.049	0.022	0.003	0.048	0.058	-0.019	0.007	-0.042	0	0.062	-0.055	0	-0.035
Emotional	0.04	-0.034	-0.009	0.141	-0.057	0.081	-0.045	-0.038	-0.017	0.009	-0.005	-0.037	0.067	0.074	-0.035	0

Note: Green shade = positive correlations, red shade = negative correlations, intensity of shade corresponds to strength of correlation

Table 2: Residual correlations in the unidimensional model – inpatient sample

	Essential char	Relation-ship	Unique person	Biopsycho-social	Communi-cation	Integration	Teamwork	Access	Coordi-nation	Safety	Information	Involvement	Family	Empower-ment	Physical	Emotional
Essential char	0	0.034	-0.02	-0.062	0.023	-0.035	0.045	0.04	-0.005	-0.035	-0.067	0.004	-0.074	-0.068	0.026	0.02
Relationship	0.034	0	0.001	-0.078	0.035	-0.089	0.059	-0.021	0.016	-0.077	-0.021	0.05	-0.107	-0.061	0.021	-0.018
Unique person	-0.02	0.001	0	0.019	0.003	0.014	-0.02	0.012	-0.018	0.023	0.007	0.025	0.027	0.027	0.007	-0.048
Biopsychosocial	-0.062	-0.078	0.019	0	-0.047	0.247	-0.093	-0.06	-0.012	0.121	0.098	-0.039	0.217	0.263	-0.127	0.204
Communication	0.023	0.035	0.003	-0.047	0	-0.056	0.005	-0.011	0.014	-0.026	0.017	-0.001	-0.06	-0.047	0.032	-0.032
Integration	-0.035	-0.089	0.014	0.247	-0.056	0	-0.069	-0.05	0.061	0.167	0.031	-0.052	0.278	0.22	-0.065	0.167
Teamwork	0.045	0.059	-0.02	-0.093	0.005	-0.069	0	0.07	0.015	-0.031	-0.024	-0.025	-0.034	-0.072	0.046	-0.071
Access	0.04	-0.021	0.012	-0.06	-0.011	-0.05	0.07	0	0.031	-0.036	-0.05	0.002	-0.038	-0.05	0.075	-0.019
Coordination	-0.005	0.016	-0.018	-0.012	0.014	0.061	0.015	0.031	0	-0.039	0.045	-0.03	0.043	-0.031	-0.036	0
Safety	-0.035	-0.077	0.023	0.121	-0.026	0.167	-0.031	-0.036	-0.039	0	0.07	-0.013	0.23	0.159	-0.057	0.035
Information	-0.067	-0.021	0.007	0.098	0.017	0.031	-0.024	-0.05	0.045	0.07	0	0.006	0.127	0.066	-0.045	0.004
Involvement	0.004	0.05	0.025	-0.039	-0.001	-0.052	-0.025	0.002	-0.03	-0.013	0.006	0	-0.041	-0.061	0.011	-0.006
Family	-0.074	-0.107	0.027	0.217	-0.06	0.278	-0.034	-0.038	0.043	0.23	0.127	-0.041	0	0.218	-0.059	0.107
Empowerment	-0.068	-0.061	0.027	0.263	-0.047	0.22	-0.072	-0.05	-0.031	0.159	0.066	-0.061	0.218	0	-0.1	0.1
Physical	0.026	0.021	0.007	-0.127	0.032	-0.065	0.046	0.075	-0.036	-0.057	-0.045	0.011	-0.059	-0.1	0	-0.061
Emotional	0.02	-0.018	-0.048	0.204	-0.032	0.167	-0.071	-0.019	0	0.035	0.004	-0.006	0.107	0.1	-0.061	0

Note: Green shade = positive correlations, red shade = negative correlations, intensity of shade corresponds to strength of correlation

Table 3: Modification indices in the unidimensional model – outpatient sample

lhs	op	rhs	mi	epc	sepc.lv	sepc.all	sepc.nox
Biopsychosocial	~~	Integration	57.9	0.745	0.745	0.300	0.300
Biopsychosocial	~~	Emotional	55.3	0.426	0.426	0.288	0.288
Integration	~~	Family	47.4	0.915	0.915	0.308	0.308
Integration	~~	Empowerment	45.9	0.712	0.712	0.279	0.279
Essential char	~~	Relationship	44.7	0.124	0.124	0.257	0.257
Biopsychosocial	~~	Family	42.3	0.660	0.660	0.281	0.281
Biopsychosocial	~~	Empowerment	38.1	0.486	0.486	0.241	0.241
Family	~~	Empowerment	37.8	0.671	0.671	0.278	0.278
Teamwork	~~	Access	34.8	0.236	0.236	0.218	0.218
Coordination	~~	Information	33.6	0.288	0.288	0.207	0.207
Safety	~~	Family	30.6	0.609	0.609	0.250	0.250
Information	~~	Empowerment	28.7	0.347	0.347	0.205	0.205
Relationship	~~	Biopsychosocial	27.6	-0.188	-0.188	-0.195	-0.195
Relationship	~~	Empowerment	27.3	-0.202	-0.202	-0.205	-0.205
Involvement	~~	Family	27.0	-0.309	-0.309	-0.231	-0.231
Communication	~~	Family	25.3	-0.261	-0.261	-0.220	-0.220
Biopsychosocial	~~	Safety	24.4	0.389	0.389	0.191	0.191
Biopsychosocial	~~	Communication	24.3	-0.178	-0.178	-0.180	-0.180
Essential char	~~	Empowerment	23.5	-0.197	-0.197	-0.194	-0.194
Essential char	~~	Family	23.4	-0.258	-0.258	-0.218	-0.218
Essential char	~~	Emotional	20.9	0.138	0.138	0.185	0.185
Relationship	~~	Integration	19.5	-0.214	-0.214	-0.176	-0.176
Essential char	~~	Integration	18.6	-0.219	-0.219	-0.176	-0.176
Communication	~~	Emotional	18.2	-0.124	-0.124	-0.166	-0.166
Relationship	~~	Safety	17.2	-0.161	-0.161	-0.161	-0.161
Relationship	~~	Physical	16.8	0.146	0.146	0.208	0.208
Integration	~~	Safety	16.2	0.427	0.427	0.166	0.166
Safety	~~	Empowerment	15.7	0.334	0.334	0.160	0.160
Relationship	~~	Involvement	14.7	0.079	0.079	0.143	0.143
Communication	~~	Involvement	14.7	0.079	0.079	0.141	0.141
Unique person	~~	Involvement	14.4	0.093	0.093	0.149	0.149
Information	~~	Family	14.0	0.317	0.317	0.161	0.161
Integration	~~	Involvement	13.2	-0.207	-0.207	-0.146	-0.146
Teamwork	~~	Coordination	12.8	0.137	0.137	0.124	0.124
Information	~~	Involvement	12.8	-0.123	-0.123	-0.131	-0.131
Unique person	~~	Teamwork	12.3	-0.094	-0.094	-0.129	-0.129
Essential char	~~	Biopsychosocial	12.1	-0.130	-0.130	-0.132	-0.132
Communication	~~	Integration	12.0	-0.171	-0.171	-0.136	-0.136
Essential char	~~	Coordination	10.9	-0.102	-0.102	-0.123	-0.123
Safety	~~	Involvement	10.6	-0.147	-0.147	-0.127	-0.127
Involvement	~~	Empowerment	10.2	-0.145	-0.145	-0.126	-0.126
Relationship	~~	Family	10.2	-0.162	-0.162	-0.141	-0.141

lhs = left hand side; rhs = right hand side; mi = modification indice; epc = expected parameter change; sepc.lv = only standardizing the latent variables; sepc.al = standardizing all variables; sepc.nox = standardizing all but exogenous observed variables

Table 4: Modification indices in the unidimensional model – inpatient sample

lhs	op	rhs	mi	epc	sepc.lv	sepc.all	sepc.nox
Biopsychosocial	~~	Emotional	75.6	0.574	0.574	0.345	0.345
Integration	~~	Family	73.3	1.056	1.056	0.375	0.375
Biopsychosocial	~~	Empowerment	68.3	0.769	0.769	0.342	0.342
Biopsychosocial	~~	Integration	66.2	0.849	0.849	0.334	0.334
Biopsychosocial	~~	Family	59.9	0.777	0.777	0.323	0.323
Safety	~~	Family	54.8	0.721	0.721	0.317	0.317
Relationship	~~	Family	44.2	-0.310	-0.310	-0.284	-0.284
Integration	~~	Empowerment	42.4	0.747	0.747	0.284	0.284
Family	~~	Empowerment	40.7	0.707	0.707	0.284	0.284
Integration	~~	Emotional	36.9	0.501	0.501	0.257	0.257
Relationship	~~	Integration	32.1	-0.273	-0.273	-0.236	-0.236
Safety	~~	Empowerment	29.8	0.485	0.485	0.228	0.228
Essential char	~~	Family	27.7	-0.263	-0.263	-0.229	-0.229
Integration	~~	Safety	27.4	0.529	0.529	0.220	0.220
Biopsychosocial	~~	Physical	26.4	-0.253	-0.253	-0.209	-0.209
Information	~~	Family	26.1	0.412	0.412	0.214	0.214
Biopsychosocial	~~	Teamwork	25.1	-0.227	-0.227	-0.190	-0.190
Relationship	~~	Biopsychosocial	23.5	-0.186	-0.186	-0.188	-0.188
Essential char	~~	Information	23.1	-0.155	-0.155	-0.186	-0.186
Relationship	~~	Safety	22.0	-0.172	-0.172	-0.185	-0.185
Biopsychosocial	~~	Safety	21.6	0.375	0.375	0.183	0.183
Teamwork	~~	Access	19.7	0.120	0.120	0.166	0.166
Teamwork	~~	Emotional	19.2	-0.157	-0.157	-0.173	-0.173
Biopsychosocial	~~	Information	18.9	0.290	0.290	0.166	0.166
Relationship	~~	Teamwork	18.1	0.087	0.087	0.160	0.160
Family	~~	Emotional	17.3	0.331	0.331	0.180	0.180
Relationship	~~	Involvement	15.9	0.092	0.092	0.154	0.154
Essential char	~~	Biopsychosocial	15.3	-0.161	-0.161	-0.155	-0.155
Essential char	~~	Empowerment	14.4	-0.173	-0.173	-0.160	-0.160
Access	~~	Physical	13.8	0.111	0.111	0.150	0.150
Essential char	~~	Teamwork	13.4	0.081	0.081	0.141	0.141
Empowerment	~~	Physical	13.0	-0.197	-0.197	-0.157	-0.157
Integration	~~	Teamwork	12.4	-0.200	-0.200	-0.144	-0.144
Integration	~~	Involvement	11.6	-0.217	-0.217	-0.142	-0.142
Unique person	~~	Emotional	11.4	-0.125	-0.125	-0.140	-0.140
Empowerment	~~	Emotional	10.9	0.243	0.243	0.141	0.141
Teamwork	~~	Empowerment	10.8	-0.164	-0.164	-0.133	-0.133
Communication	~~	Family	10.4	-0.157	-0.157	-0.138	-0.138
Essential char	~~	Access	10.3	0.080	0.080	0.127	0.127

lhs = left hand side; rhs = right hand side; mi = modification indice; epc = expected parameter change; sepc.lv = only standardizing the latent variables; sepc.al = standardizing all variables; sepc.nox = standardizing all but exogenous observed variables

Given that the model fit indices indicated a slight misfit in the unidimensional model, based on a combination of residual correlations, model fit indices and theoretical considerations to refine the model in an exploratory manner by incorporating residual correlations between items.

For both samples, we identified items with high residual correlations and for which modification indices suggested a substantial improvement in model fit when residual correlations were added to the model. We then assessed the conceptual coherence of these modifications to ensure their theoretical plausibility. After incorporating a correlation into the modified model, we reanalyzed the model fit indices and repeated this process until no clear pattern emerged across both samples.

In the modified model, four additional correlations were added:

(1) Correlation between the item for "Biopsychosocial perspective" and the item for "Emotional support" (standardized residual correlation (SRC) 0.141 for outpatients and 0.204 for inpatients): From a theoretical perspective this is justified since the biopsychosocial perspective describes applying a holistic view. Taking into account "the entire personal life" inherently includes addressing their emotional well-being, thereby increasing the likelihood of acknowledging fears and concerns.
(2) Correlation between "Integration of medical and non-medical care" and "Involvement of family and friends" (SRC 0.249 outpatients and 0.278 inpatients): Both items describe a proactive behavior by the healthcare professionals to inform about aspects that can be integrated in addition to the primary biomedical treatment.
(3) correlation between "Biopsychosocial perspective" and "Integration of medical and non-medical care" (SRC 0.219 outpatients and 0.247 inpatients): Both items mention spiritual and religious aspects.
(4) Correlation between "Biopsychosocial perspective" and "Involvement of family and friends" (SRC 0.194 outpatients and 0.217 inpatients): Again, both items include aspects considering the family.

The revised model demonstrated improved fit indices (see Table 5), with all but one index falling within the recommended range. Although one fit index remained slightly above the optimal threshold, the overall improvement suggests that the postulated unidimensional model fits the data set well, although the presence of a few residual correlations is possible. As these modifications are of exploratory character, future research is needed to confirm that the modified model actually fits in other samples as well and does not just reflect particular characteristics of this sample.

Table 5: Model fit indices for modified models

	Chi square			RMSEA	SRMR	TLI	CFI	AIC	BIC
	Chi²	df	P value						
**Recommendation**			>.05	≤ .06	<.08	≥ .90	≥ .90	Smallest value	
**Outpatient sample**									
Original model	843	104	<0.001	0.081	0.066	0.877	0.894	43910	44149
Modified model	639	100	<0.001	0.070	0.059	0.907	0.922	43714	43973
**Inpatient sample**									
Original model	856	104	<0.001	0.088	0.073	0.865	0.883	39789	40022
Modified model	604	100	<0.001	0.074	0.065	0.906	0.922	39545	39797

Abbreviations: df = degrees of freedom; RMSEA = Root Mean Square Error of Approximation; SRMR = Standardized Root Mean Square Residual; TLI = Tucker-Lewis Index; CFI = Comparative Fit Index; AIC = Akaike information criterion; BIC = Bayesian information criterion
