# Supplementary material for: Development and Psychometric Testing of EPAT‐16: A Short and Valid Measure for Patient‐Centeredness From the Patient's Perspective
Source: Health Expect. 2025 May 20;28(3):e70296. doi: 10.1111/hex.70296 (PMC12090203; doi:10.1111/hex.70296)
Supplement: Supplementary file 5 — Appendix 5 Descriptive statistics of EPAT‐16 sum score per group. [file HEX-28-e70296-s001.pdf]

Appendix 5: Descriptive statistics of EPAT-16 sum score per group

Article: Development and psychometric testing of EPAT-16: A short and valid measure for patient-centeredness from the patient's perspective

	Outpatient sample					Inpatient sample				
	All	Card	Cancer	Mus	Ment	All	Card	Cancer	Mus	Ment
**M**	72.6	70.9	73.5	68.3	76.6	73.6	72.8	77.5	66.9	71.0
**SD**	15.7	15.3	14.7	18.0	13.7	15.2	14.1	13.4	18.2	16.5
**Percentiles**										
5	42	45	46	35	47	46	50	53	31	38
10	50	51	55	42	59	53	54	59	36	48
15	56	55	58	47	64	58	59	63	46	54
20	60	58	61	52	67	62	61	65	50	57
25	63	61	64	56	69	64	64	69	55	63
30	66	63	66	59	71	67	66	71	60	65
35	68	65	68	64	74	70	68	74	62	67
40	70	68	71	66	76	72	70	76	64	70
45	73	70	73	68	78	74	72	78	66	72
50	75	72	75	71	79	76	73	80	69	74
55	77	74	77	74	81	78	76	81	73	75
60	79	77	79	77	82	80	78	83	76	77
65	81	79	80	79	84	82	79	85	78	80
70	83	81	83	80	86	84	82	87	80	82
75	85	83	85	82	87	86	84	88	82	84
80	87	85	87	85	89	88	86	90	84	86
85	89	88	90	87	90	90	88	91	85	88
90	91	90	92	90	92	92	91	93	89	90
95	94	93	95	93	94	94	93	95	90	92
100	96	96	96	96	96	96	96	96	96	95

Abbreviations: Card = Cardiovascular diseases; Mus = Muscoloskeletal diseases; Ment = Mental disorders; M = mean; SD = standard deviation
